# Barriers, facilitators, and proposals for improvement in the implementation of a collaborative care program for depression: a qualitative study of primary care physicians and nurses

**DOI:** 10.1186/s12913-022-07872-z

**Published:** 2022-04-05

**Authors:** Enric Aragonès, Germán López-Cortacans, Narcís Cardoner, Catarina Tomé-Pires, Daniel Porta-Casteràs, Diego Palao, the INDI·I Research Team, Eva Bellerino, Myriam Cavero, Eva Aguilar, Marta Subirà, Antonia Caballero, Pilar Casaus, José Antonio Monreal, Víctor Pérez-Sola, Miquel Cirera, Maite Loren, Laura Palacios

**Affiliations:** 1 Primary Care Area Camp de Tarragona, Catalan Health Institute, Carrer dels Horts, 6, 43120 Constantí, Tarragona, Spain; 2 Primary Care Research Institute IDIAP Jordi Gol, Barcelona, Spain; 3 Mental Health Department, University Hospital Parc Taulí, Sabadell, Spain; 4 Department of Psychiatry and Legal Medicine, Autonomous University of Barcelona, Barcelona, Spain; 5 Biomedical Research Networking Center Consortium on Mental Health (CIBERSAM), Madrid, Spain; 6 Psychology Research Center CIP, Autonomous University of Lisbon, Lisbon, Portugal

**Keywords:** Depressive disorder, Primary health care, Health plan implementation, Disease management, Qualitative research, Focus groups

## Abstract

**Background:**

Primary care plays a central role in the treatment of depression. Nonetheless, shortcomings in its management and suboptimal outcomes have been identified. Collaborative care models improve processes for the management of depressive disorders and associated outcomes. We developed a strategy to implement the INDI collaborative care program for the management of depression in primary health care centers across Catalonia. The aim of this qualitative study was to evaluate a trial implementation of the program to identify barriers, facilitators, and proposals for improvement.

**Methods:**

One year after the implementation of the INDI program in 18 public primary health care centers we performed a qualitative study in which the opinions and experiences of 23 primary care doctors and nurses from the participating centers were explored in focus groups. We performed thematic content analysis of the focus group transcripts.

**Results:**

The results were organized into three categories: facilitators, barriers, and proposals for improvement as perceived by the health care professionals involved. The most important facilitator identified was the perception that the INDI collaborative care program could be a useful tool for reorganizing processes and improving the management of depression in primary care, currently viewed as deficient. The main barriers identified were of an organizational nature: heavy workloads, lack of time, high staff turnover and shortages, and competing demands. Additional obstacles were inertia and resistance to change among health care professionals. Proposals for improvement included institutional buy-in to guarantee enduring support and the organizational changes needed for successful implementation.

**Conclusions:**

The INDI program is perceived as a useful, viable program for improving the management of depression in primary care. Uptake by primary care centers and health care professionals, however, was poor. The identification and analysis of barriers and facilitators will help refine the strategy to achieve successful, widespread implementation.

**Trial registration:**

ClinicalTrials.gov identifier: NCT03285659; Registered 18th September, 2017.

## Introduction

Depression is a critical public health problem that has a significant impact on patients, the people around them, and society as a whole [1]. According to the ESEMeD-Spain survey, 10.6% of non-institutionalized Spanish adults have had major depressive symptoms at some point in their life and 4% have had episodes in the past 12 months [2]. The cost of depression in Spain was estimated at €10,763 million in 2010, with indirect costs related to work productivity losses and disability accounting for 45% of the total [3].

The most common mental health disorders can be managed effectively and efficiently in primary care [4]. This includes depression, but shortcomings in diagnosis, treatment, and follow-up often lead to unsatisfactory clinical outcomes [5, 6].

Collaborative care models, which are based on the chronic care model [7] can improve the management of chronic illnesses, such as depression. These models are shared care plans where health professionals from different levels of care coordinate the management of patients within a common structure that includes systematic planning of care provision and follow-up [8]. Collaborative care models improve processes for the management of depression and result in better clinical outcomes [9]. The evidence to date indicates that their wider implementation should be recommended, particularly in primary care settings [10, 11].

Difficulties, however, are encountered when complex models such as these are integrated into clinical practice [12, 13]. Our team designed and implemented a strategy for the widescale implementation of a multicomponent collaborative care program (INDI) designed to improve the management of depression in primary care centers within the Catalan public health system [14]. Results from a clinical trial assessing the efficacy of the program indicated that its implementation would be beneficial [15].

The aim of this study was to evaluate a trial implementation of the INDI collaborative care program in real-life clinical practice using a qualitative design in which we explored the opinions and experiences of the primary care doctors and nurses involved. We employed a comprehensive approach in which in addition to evaluating the implementation, we studied the influence of program characteristics and contextual factors on the process. We focused on perceived barriers to and facilitators of successful implementation in daily practice and proposals for improvement.

## Methods

### Design overview

One year after launching our strategy to promote the implementation of the INDI collaborative care program for the management of depression in primary care, we undertook an exploratory qualitative study using focus groups to explore the perceptions of the primary care doctors and nurses from the participating centers.

The study protocol was approved by the Clinical Research Ethics Committee of the Jordi Gol Primary Care Research Institute (IDIAP) in Barcelona (P17/077; 15/03/2017). Informed consent was obtained from all participants. The study was registered at ClinicalTrials.gov (NCT03285659; 18/09/2017). It was conducted and reported in accordance with the consolidated criteria for reporting qualitative studies (COREQ) [16].

### Setting

The program was implemented in primary care centers in two health care districts: Tarragona-Valls, located in southern Catalonia, and Vallès Occidental, located in the metropolitan region of Barcelona. Twelve centers, with 146 doctors, 136 nurses, and an assigned population of 209,591 people, participated in the first district, while six centers, with 88 doctors, 81 nurses, and an assigned population of 144,884 people, participated in the second. As the goal of the study was to test the program in real-life conditions, no exclusion criteria were applied to either centers or health care professionals.

### Participants

Primary care doctors and nurses from the centers in both study districts were recruited to the focus groups. Recruitment was purposive. We decided to separate doctors and nurses into different groups to encourage frank and open discussions and prevent any one group from dominating the discussion. Doctors, for example, might be expected to have more elaborated opinions on how depression should be managed. The obvious disadvantage of separating the groups was that it did not allow for the sharing and exploration of different perspectives. Participants had to have had direct experience in the management of patients with depression and be familiar with the key principles and characteristics of the INDI program [14, 15], regardless of the level of uptake at their center. They did not receive any compensation for their participation. Their characteristics are summarized in Table 1.

**Table 1:** Sociodemographic and professional characteristics of focus groups participants

Code	Focus group^a^	Profession	Gender	Age (years)	Years working in primary care
TD1	TD	Family doctor	Female	49	14
TD2	TD	Family doctor	Male	52	24
TD3	TD	Family doctor	Female	41	12
TD4	TD	Family doctor	Female	58	31
TD5	TD	Family doctor	Female	45	16
TD6	TD	Family doctor^b^	Female	43	15
TN1	TN	Primary care nurse	Female	60	20
TN2	TN	Primary care nurse	Female	47	15
TN3	TN	Primary care nurse	Female	57	28
TN4	TN	Primary care nurse	Male	58	25
TN5	TN	Primary care nurse	Female	50	23
TN6	TN	Primary care nurse	Female	37	15
SD1	SD	Family doctor^b^	Female	42	14
SD2	SD	Family doctor	Male	50	22
SD3	SD	Family doctor	Female	56	30
SD4	SD	Family doctor	Female	55	29
SD5	SD	Family doctor	Male	38	11
SN1	SN	Primary care nurse	Male	48	25
SN2	SN	Primary care nurse	Female	54	29
SN3	SN	Primary care nurse	Female	43	20
SN4	SN	Primary care nurse	Female	44	18
SN5	SN	Primary care nurse	Female	58	28
SN6	SN	Primary care nurse	Female	48	25

^a^Focus groups performed: TD (family doctor in Tarragona); TN (primary care nurse in Tarragona); SD (family doctor in Sabadell); SN (primary care nurse in Sabadell)

^b^professional with a special interest in mental health (‘more than average compared to my colleagues’)

### Program implementation

INDI is a multicomponent collaborative care program designed to meet the characteristics and needs of the Catalan public primary care health system [15]. Its goal is to improve the management of depression and clinical outcomes. Its cost-effectiveness was evaluated in a randomized controlled trial, which demonstrated a favorable incremental cost-effectiveness ratio and concluded that its implementation could benefit both patients and the health care system [15, 17].

The PARIHS (Promoting Action on Research Implementation in Health Services) framework was used to design and deploy the strategy for transferring the intervention to clinical practice [18]. A more detailed description of this strategy has been published elsewhere [14]. Its main components and those of the INDI program are summarized in Table 2.

**Table 2:** Main components of INDI program for the management of depression in primary care and the implementation strategy based on the PARIHS (Promoting Action on Research Implementation in Health Services) framework

**INDI program**	
Redefinition of practitioner roles and care pathways within the primary care team	
Optimized management of depression. Interactive computerized clinical guideline to support patient monitoring and decision-making	
Introduction of the figure of care manager assigned to primary care nurses	
Patient psychoeducation program	
Improved liaison between primary care and psychiatry services. Shared care	
**Implementation strategy**	
Compilation and analysis of implicit evidence (knowledge and reflections of health care professionals and patients targeted by the program) and explicit evidence (clinical trials, economic evaluations, meta-analyses)	
Analysis of institutional setting and characteristics (e.g., organizational aspects, innovation culture, quality, continuous professional development) that could negatively or positively affect the implementation of the program	
Internal facilitators − Regional leaders of INDI program linked to clinical management in both health care districts as a driver for local implementation − Leading health care professionals to champion the program at each primary care center	
External facilitators provided by research team: online training for health care professionals, support and guidance, evaluation, feedback, local adaptation of intervention, accreditation of centers and practitioners, interinstitutional coordination.	

### Data collection

Four focus groups were held: one each with primary care doctors (TD) and nurses (TN) in the Tarragona-Valls district and one each with primary care doctors (SD) and nurses (SN) in the Vallès Occidental district.

The script for the focus groups was prepared in accordance with the study objectives. Rather than using predefined codes or evaluation areas, we prepared a short, simple script that would allow us to explore emerging categories and ideas based on the participants’ perceptions and experiences (Table 3). Each group had a moderator (GLC in Tarragona and DPC in Sabadell) and an observer (CTP in Tarragona and NC in Sabadell) who took notes during the session, paying special attention to non-verbal language. Both the moderators and observers are trained certified qualitative researchers [19], were members of the team responsible for promoting the INDI intervention, and had participated in meetings and training sessions attended by the focus group participants. Their credentials and affiliations are listed in the authorship section of the article.

**Table 3:** Summary of focus group script

**−**Experience with and opinion of the implementation of the INDI program, in whole or in part, in daily practice and at the primary health care center	
**−** Factors that influence the implementation of changes promoted by the INDI program (difficulties and barriers, and facilitators)	
**−** Identification and prioritization of actions or factors that could improve the implementation of the INDI program and make it more effective and useful	

The focus groups were held in meeting rooms of the territorial delegations of the Catalan Institute of Health in Tarragona and Sabadell and lasted approximately 90 min each. The participants were familiar with the general objectives of the study and the relationship between the group moderators and the research team. All sessions were audio-recorded and transcribed in full.

Field notes on direct observations and interactions between researchers and health care professionals at the participating centers were taken throughout the implementation process to complement other data. These notes were useful for contextualizing and better understanding data obtained in the focus groups.

### Analysis

The focus group transcripts and field notes were analyzed by themes [20]. Content analysis was used to highlight meanings within the text to describe and/or interpret the themes that emerged. The steps in the above analyses were (a) careful reading of transcripts and field notes, (b) identification of relevant topics and texts, (c) breaking down of text into units of meaning, (d) coding of texts using a mix of predefined and emerging codes, (e) creation of categories that grouped together analogous codes according to pre-established analytical criteria, (f) analysis of points of agreement and disagreement, and (h) triangulation of results [21]. The data were summarized, grouped into conceptual categories, and analyzed using standard qualitative research techniques in collaboration with the study researchers (GLC, CTP, EA). No software was used to process the data. Participants were not asked for their opinions on the findings.

## Results

### Participation

Eleven doctors with a mean (SD) age of 48.1 (6.7) years and 12 nurses with a mean age of 50.3 (7.1) years participated in the focus groups in the two districts. Their demographic and professional characteristics are summarized in Table 1.

### Results of analysis

The results of the analysis were organized into three categories according to the study objectives: (1) difficulties and barriers to the effective implementation of the INDI program, (2) facilitators that could drive its implementation, and (3) suggestions from the health care professionals involved on how to improve the implementation and effectiveness of the program for the management of depression in clinical practice. In Table 4, within these categories we have classified the results according to whether they were contextual (e.g., healthcare organization, professionals, or patients), whether they concerned the strategy and procedures carried out for implementation, or whether they could be attributed to the INDI program itself.

**Table 4:** Summary of focus group thematic analysis results

**Obstacles**	
*Organization*	Lack of time
	Heavy workloads
	Staff shortages
	High staff turnover
	Changing, unstable leadership
	Diversity of teams
	Multiplicity of programs and initiatives (competing demands)
	Perceived lack of institutional buy-in
*Health care professionals and patients*	Apprehension, insecurity, lack of training/qualification among nurses
	Resistance among nurses to adopt new role
	Resistance among patients (to be managed by a nurse)
	Distrust of nurses’ work among doctors
	Difficulty coordinating shared work between doctors and nurses
	Routines, resistance to change among health care professionals
*Implementation strategy*	Top-down implementation, generating resistance among some professionals
	Lack of clarity in the definition of the care manager role
	Limitations in training program
	Difficulties coordinating shared care among primary care teams and psychiatry services. Compartmentalized work, not shared
*INDI program*	Complexity of the program
	Limitations in reliability of scales
**Facilitators**	
*General aspects*	Prevalence and importance of depression in primary care
	Current shortcomings in management of depression and need for improvement
	Recognition that depression should be managed by primary care
*Implementation strategy*	Useful, well-received training program
*INDI program*	Recognition, reinforcement, structuring, and systematization of the role of nurses
	Systematic use of guidelines and scales to facilitate structured management
	Greater access to and support from mental health specialists
**Proposals**	
*Healthcare institution*	Clear buy-in from institution
	Actions and measures to facilitate organizational changes required
	Inclusion of program in target payment system
	Involvement of health care professionals implementation decisions and design
	Stable doctor-nurse teams
*Implementation strategy*	Continued professional development for health care professionals
	Reinforcement of practical aspects of training
	Combination of online and face-to-face training sessions
	“Local” expert health care professionals to support teams
	Integration of depression management into community care
	Closer liaisons between primary care and mental health services
*INDI program*	More clearly defined roles for nurses (care managers)
	Development of a clear concept of shared care between primary care and psychiatry services

The INDI program was largely perceived as an opportunity to restructure and improve the management of depression, which was recognized as a common and important condition whose current management presented shortcomings and difficulties. Most of the health care professionals interviewed were unsatisfied with the implementation process and results of the program. They felt that it had not been implemented systematically or on a widespread scale, although they did recognize that some of its components had been partly introduced.

“I see it as an opportunity for improvement, other things we can do as nurses, a means for reinforcing the role of nurses” (TD2)

“Minimum implementation. Some doctors and a few nurses have done it, but not many” (SD2)

### Barriers to implementation

The main barriers to the successful implementation of the INDI program were of an organizational nature and were viewed by the doctors and nurses as being outside their area of responsibility: work overload, a lack of time for activities other than meeting patient demands, and staff shortages and turnover rates that make it difficult, if not impossible, to work as a team.

“... We’re run off our feet in primary care, [...] so when a new task comes in, whether it’s related to mental health or something else, we tend to say no, because of the volume of work” (SD2)

“... it’s been a complicated time. There aren’t enough doctors...” (TN3)

“... there has been a lot of turnover: first you work with one person, then with another, there’s very little stability” (SN1)

Reference was also made to the fact that numerous projects, programs, and training activities were launched at the same time, each competing for the doctors and nurses’ time, attention, and effort. This creates resistance to accepting new initiatives, which are viewed as more work. The field notes included various references to the perception among the health care professionals that the health organization had not prioritized the implementation of the INDI program.

“... we have daily training sessions on the ARES [a new system for recording nursing activities], sessions on how to manage chronic illnesses, the patient safety course, I mean, we have meetings and courses every day…” (SN1)

The perception that the program had been imposed top-down, without consultation, may have generated feelings of resistance.

“... the fact that it came straight from above was seen in a negative light...” (TD6)

The nurses, who have a key role in the INDI program, said that they were motivated by the project, but also mentioned that their lack of knowledge, skills, and training in this area made them feel uneasy and insecure. They also had the impression that the goal of the program was to increase the responsibilities of nurses with the spurious aim of reducing the doctors’ workload

“I think that training is missing, because the apprehension that nurses feel about dealing with mental health issues is due to a lack of training” (SN2)

Mention was also made of shortcomings in coordination between doctors and nurses. Both groups agreed that it was not usual for nurses to follow-up on depression in their visits with patients. On the one hand, patients showed resistance, while on the other, doctors were slow to change their work routines by incorporating the program’s recommendations on shared care. Some doctors expressed feelings of distrust towards nurses. They also mentioned that nurses had not become sufficiently involved and that it was difficult to accept managing depression as part of their role.

“... integration of the concept of team is missing when it comes to managing this disease” (TD4)

“Do you think that doctors have done what they should? [...] no patients have been shared with nurses” (TN3)

“I have also encountered some resistance from some nurses, hey ... I mean, apart from the inertia that we [doctors] have of “I’ll look after everything ...” (TD4)

Other barriers identified were coordination difficulties between primary care and psychiatry, a lack of efficient communication procedures, and poorly defined tasks and responsibilities at each level of care. A mental health support program requiring psychiatrists and psychologists to be physically present at some of the primary care centers was implemented at the same time as the INDI program. This greater access to specialist support facilitated the coordination and integration of tasks, but it also made it easier to hand over responsibilities for mental health care provision to these specialists.

### Facilitators

Our findings show that primary care doctors and nurses largely recognize the significant impact of emotional problems in routine primary care practice and their link to general health. They also perceived deficiencies in the management of mental health disorders. Because the INDI program focuses on perceived needs, these perceptions should conceptually facilitate its implementation.

“...mental health has always been neglected... And we see this in primary care: 90% of what we deal with is related to emotional issues” (SN2)

There was a call for the primary care system to take on an increasing role in managing patients with depression and other mental health disorders.

“... the health system is increasingly transferring more and more mental health competencies to primary care [...]. We are in a process of change [...]. Yes, we are saturated, yes, we have heavy workloads, but we want to take on this role” (SN2)

Some nurses were in favor of working more with mental health. They were motivated and willing and saw the project as an opportunity to take on a greater role by working together with primary care doctors and mental health specialists. They were of the opinion that the INDI program provides useful tools and the means to build on work that is already being done, often in an unstructured way: follow-up and control, detection of episodes, treatment adherence, and the combined management of mental health illnesses and somatic comorbidities.

“These patients shouldn’t have to go to the doctor often. They should be managed by nurses” (TN2)

“... in some way, it built on everything that was being done […]. But I must say that mental health is dealt with in combination with other diseases in our practice, because it’s not a separate thing, everything is mixed up and everything is treated at the same time.” (SN3)

It was largely agreed that an interdisciplinary approach to mental health care provision was a good idea. Some participants also mentioned that when the program had been implemented, even partially, the doctor-nurse dyad had worked well.

“Some people are implementing the program [...], but more because certain nurses are personally motivated and work well with the doctors than because of a widespread interest within the team” (SN2)

Some doctors considered that nurses were equipped to follow up on patients with depression; they said that they knew how to do this, were committed, knew the patients well, had their trust, and had longer visits with them than the doctors.

“... [nurses] can do this perfectly, and sometimes they are more of a “psychologist” than we are [doctors], they are the ones managing the patients ... they also have long visits in which they can go into things in more depth, in a more relaxed environment, than we can” (SD4)

Systematic use of questionnaires, such as the suicide risk screening questionnaire and the Patient Health Questionnaire (PHQ-9) to quantify depression severity and monitor symptoms and response to treatment over time, was uncommon before recommended by the INDI program. The health care professionals were of the opinion that these questionnaires were now easy to administer in routine practice as they had been included in the electronic medical record system. They recognized their usefulness as a clinical monitoring tool.

“As for indicators, the PHQ-9 and changes to diagnostic practices, yes, this has happened in the last 2 years, quite a lot, a lot, has been done. Both the suicide risk questionnaire and the PHQ-9 have been used quite a lot” (SN3)

The relationship with psychiatry and psychology teams was generally perceived as good and those interviewed mentioned that they appreciated their accessibility and support.

“And the relationship with the psychologist/psychiatrist at the center is fantastic too. We didn't have a relationship and now we do, I have to give you that” (TD1)

“... we do, doctors and nurses participate. If there are specific issues to deal with, even the social worker comes” (SD1)

### Proposals for improvement

Buy-in from the Catalan Health Institute and primary health care center management is critical to the effective implementation of the guidelines and tools contemplated within the INDI program. Enduring institutional support must be guaranteed alongside organizational changes needed to improve the functioning of primary care teams and their relationship with mental health services.

“If management at each center was committed, said 'I’m going to do it, I’m going to implement this in my center' and they look for a model to follow and with support from mental health services [...] we’re in” (TN1)

Clear institutional buy-in was considered a key factor to the success of any new program; this could be achieved by including the program, with specific, measurable indicators, in the institute’s target payment system (DPO, Direcció per objectius). General recommendations on the merits of a given project, by contrast, lead to heterogeneous results, as their success depends on the willingness and resources of each center and its staff.

“Question: Do you think that if the INDI program, in its current format, had been included in the DPO [target payment] system...

Answer: Yes, definitely. Sadly yes, I think this is the case. The results would be completely different” (TD5)**.**

Although the focus group participants believed that institutional and management prioritization and support were essential, they were also of the opinion that the health care professionals dealing with patients should be involved in these decisions.

“... we’re all saturated and when things are imposed from above ... it’s like 'the ball’s in your court now', and there’s no 'look, they’re looking to do this… we should do that...'. Team work and empowerment” (SN1)

Other proposals that emerged were changes to primary care organizational structures, programming of visits, and internal pathways that would facilitate collaboration between doctors and nurses and give them time to address the needs of patients with depression. One proposal for facilitating collaborative work mentioned in the focus groups and field notes was the creation of stable doctor-nurse teams (unitat bàsica assistencial, UBA) with the same patients under their care.

“... that’s why you need to improve the programming of visits, prepare a series of things so that nurses can monitor patients with depression” (TN2)

“... maybe longer special visits should be programmed, otherwise, there’s no time for psychoeducation...” (SN3)

“... we have improved by returning to the UBA [basic care unit consisting of a doctor and a nurse who share and are co-responsible for the same patients], because before that we shared our patients with all the nurses” (TD3)

Clearly defined roles and responsibilities and proper structuring of tasks and actions would build nurses’ confidence and help them overcome current resistance and fears in relation to their new role in managing patients with depression.

“... one way to do this would be to create a clinical pathway, as with any other chronic illness. You standardize it: diagnosis, questionnaire, initial diagnosis, risk of suicide, guidance, if it’s mild no drugs, if it's moderate, or whatever, drugs, nurses, number of follow-up visits” (SD1)

Community care was identified as an area where the INDI program could be implemented and progressively expanded. One advantage of this approach would be that community care nurses could apply their existing experience and know-how to help prevent and manage depression within the community.

“So many things are interlinked in the community. We could do this as part of community care” (SD1)

## Discussion

Primary care doctors and nurses recognize that mental health disorders, and depression in particular, are widespread in routine clinical practice and acknowledge that there are shortcomings in their current management. They also view the INDI program as a potentially useful tool for achieving higher-quality care. Uptake of the program, however, has been poor, both across primary care centers and among health care providers. The main barriers identified were problems perceived to be beyond the responsibility and control of the health care professionals: heavy workloads, insufficient time, staffing problems, and a lack of institutional leadership. These findings are supported in the literature [22–24].

The doctors and nurses interviewed, however, also acknowledged problems linked to their own inertia and resistance to change. Doctors on the one hand were reluctant to share responsibilities for managing patients with depression with nurses, while nurses were reluctant to take on new responsibilities. Resistance to change among health care professionals has been identified as the most common barrier to the success of collaborative care models [25, 26].

The care manager is a key figure in collaborative care models. In the INDI model, this role is assigned to primary care nurses, who, working closely with the patient’s primary care doctor, take on responsibilities in monitoring clinical course and treatment adherence and providing emotional and self-management support [27]. The figure of care manager is a novel concept in our setting and one that may prove difficult to accept or understand, as it involves profound changes to the way depression has traditionally been managed. Doctors have to relinquish responsibilities they have held for a long time, while nurse care managers have to take on new ones. An additional challenge is the need to ensure smooth, effective coordination. An explicit definition of the exact role and responsibilities of a care manager, and one that is fully understood and accepted by all parties involved, will provide care managers with the necessary confidence to exercise their new functions and doctors with clarity on the benefits of shared responsibilities. A lack of clarity on this definition has been highlighted as a major obstacle in other studies [28, 29]. It also probably partly explains the resistance encountered among the doctors and nurses in our study. Well-defined care manager roles and duties are key to the success of the program and, in view of the favorable attitudes detected among both nurses and doctors, would probably result in a more effective implementation of this role [30, 31]*.*

Complexity has also been identified as a barrier to effective implementation [32]. The INDI program is a complex, multicomponent model involving various levels of care that need to be linked via clear, coordinated pathways and procedures. This complexity, however, was not explicitly mentioned in the focus groups. We did, however, see evidence of it in the field notes taken throughout the process. Complexity is also implicit in several aspects of the program analyzed, such as difficulties with shared case management within primary care teams and between primary care teams and other levels and the fact that only some components of the program were adopted (e.g., use of questionnaires for monitoring symptoms). Similar difficulties have been reported elsewhere [33].

Our study also identified important facilitators that should be harnessed to drive successful implementation. The participants recognized the existence of shortcomings in the current management of depression and viewed the INDI program as a valuable tool that could overcome these shortcomings. Our results confirm previous reports of largely favorable attitudes among primary care practitioners towards managing depression in primary care [34, 35] and increasing nurses’ responsibilities in this area. These attitudes must be harnessed to drive effective change through clear institutional buy-in and leadership and the implementation of structural changes, but with an active bottom-up approach involving front-line professionals and local clinical leaders to champion the program. This shared leadership and vision is essential and must filter down from the highest levels to everyday practice [25, 26].

### Limitations

Our study has several limitations that should be taken into account when interpreting our findings. First, the perceptions and opinions of the small number of primary care physicians and nurses may not be representative of those who did not participate in this study. One example of this is that all the participants in our focus groups had extensive work experience. In addition, our findings cannot be directly transferred to settings or health organizations with different characteristics. Nonetheless, our focus group findings for the two health districts were similar and were also consistent with the field notes, adding strength to the validity of our results. Second, we did not interview other relevant stakeholders involved in the INDI program, such as health care managers, mentioned several times in this study, and patients, the beneficiaries of the program. Further research should explore the perspectives of all parties involved to obtain a more complete picture. Third, the INDI program focuses on depression only, but patients with depressive symptoms seen in primary care often have concomitant psychiatric disorders, chronic physical illnesses, or social problems. This should be taken into account in future implementations of the INDI collaborative care model [30, 36].

## Conclusions

This study provides useful information, based on real clinical practice, for the implementation of a collaborative care program for the improved management of depression in primary care in Catalonia. Our results provide quite detailed insights into barriers to and facilitators of the integration of a complex collaborative care program into clinical practice from the perspective of health care practitioners. A key question that remains is why uptake was low in our case. Was it the complexity of the intervention, shortcomings in the implementation strategies employed, or contextual factors? Based on the focus group findings, we believe that contextual factors related to the healthcare organization and the doctors and nurses themselves were especially decisive. Real-life implementation of collaborative care programs, however, is complex and likely to have been influenced by multiple, and often overlapping, factors from the three domains and by interactions between these factors. Consideration of all these variables is key to successful implementation.

Institutional buy-in and clear, proactive leadership are key to laying the ground for the changes required to ensure the successful implementation of the program. Inertia and resistance among practitioners must also be overcome. We have identified factors that can drive effective implementation, in particular, the general perception among front-line practitioners that the INDI program can help overcome shortcomings in the current management of depression in primary care.

## Data Availability

The data supporting the results of this study consist of audio files and transcripts of focus group meetings and are not publicly available due to the conditions set out in the consent given by participants, but are available upon reasonable request to the corresponding author.

## Abbreviations

ARES: Program for harmonization of nursing care standards
COREQ: Consolidated criteria for reporting qualitative research
DPO: Target payment system (in Catalan: *Direcció per Objectius*)
ESEMED: European Study of the Epidemiology of Mental Disorders
IDIAP: Primary Care Research Institute (in Catalan: *Institut d’Investigació en Atenció Primària*)
INDI: Interventions for Depression Improvement (acronym for the collaborative care model in this project)
INNÒBICS: Open Research at the Catalan Health Institute (in Catalan: *Innovació Oberta a l’ICS*)
PARIHS: Promoting Action on Research Implementation in Health Services
PERIS: Strategic Plan for Research and Innovation in Health (in Catalan: *Pla Estratègic de Recerca i Innovació en Salut*)
PHQ9: Patient Health Questionnaire, 9 items
